# Adsorptive Removal of Amoxicillin Using Green-Synthesized SiNH_2_@FeNP Nanocomposite: Characterization, Optimization, and Modeling

**DOI:** 10.1021/acsomega.5c02529

**Published:** 2025-06-11

**Authors:** Muhammet Yunus Pamukoğlu, Belgin Babar Yoldaş

**Affiliations:** Faculty of Engineering and Natural Sciences, Department of Environmental Engineering, 52994 Suleyman Demirel University, Isparta 32260, Turkey; Republic of Türkiye Ministry of Agriculture and Forestry, Adıyaman Research Institute for Hard-Shelled Fruits, Adıyaman 63100, Turkey

## Abstract

The increasing presence of pharmaceutical contaminants, particularly amoxicillin (AMX), in aquatic environments necessitates the development of efficient and sustainable removal strategies. In this study, the SiNH_2_@FeNP nanocomposite was synthesized via a green synthesis approach using licorice (Glycyrrhiza glabra) root extract as a reducing and stabilizing agent. The synthesized nanocomposite was comprehensively characterized using Fourier transform infrared spectroscopy, X-ray diffraction, scanning electron microscopy (SEM), and Brunauer–Emmett–Teller surface area analysis to confirm its structural and morphological features. SEM imaging revealed significant morphological changes, demonstrating a uniform FeNP distribution on the SiNH_2_ surface. The adsorptive performance of the nanocomposite was optimized using the Box-Behnken experimental design, considering key operational parameters such as pH, initial AMX concentration, and adsorbent dosage. The statistical analysis validated a quadratic model as the best fit, achieving a maximum removal efficiency of 95% under optimized conditions (pH 5.6, AMX concentration 40.79 mg/L, and adsorbent dosage 1.85 g/L). These results demonstrate the high potential of SiNH_2_@FeNP as a low-cost, eco-friendly adsorbent for pharmaceutical removal from water, providing a scalable and sustainable alternative for wastewater treatment applications. This approach represents a novel combination of green nanotechnology and statistical optimization for AMX removal.

## Graphical abstract

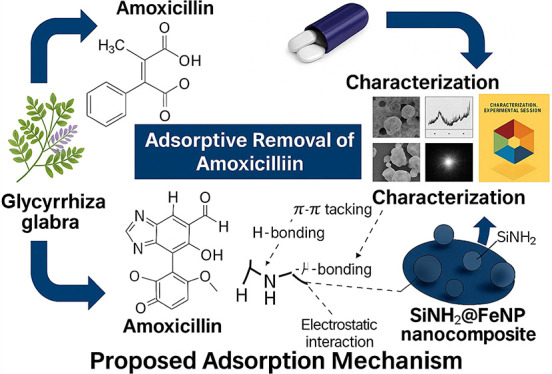

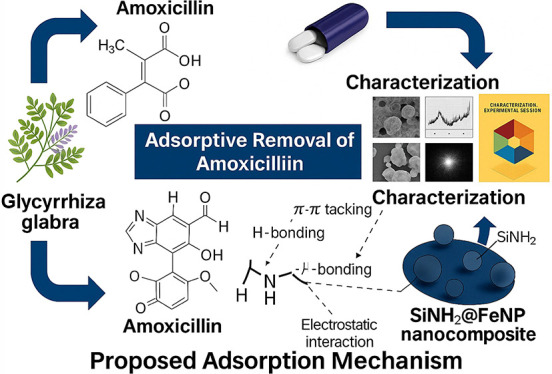

## Introduction

The term “nano,” derived from the Greek word for “dwarf,” refers to a unit of measurement that represents one-billionth. Nanotechnology plays a key role in environmental remediation, especially through nanomaterials like iron-based nanoparticles due to their high surface area, reactivity, and eco-compatibility.^1,2^ There are three primary methods for synthesizing nanoparticles: chemical, physical, and biological approaches. Among these, biological systems and microorganisms play a pivotal role in the synthesis of metal nanoparticles.^3–5^ The biological approach is preferred over physical and chemical methods due to its lower cost, simplicity, reduced use of hazardous chemicals, minimal energy consumption, and environmental friendliness.^6–8^ Green synthesis utilizes natural extracts—such as from Glycyrrhiza glabra—for reducing and stabilizing metal nanoparticles, avoiding toxic chemicals while maintaining high efficiency.^9^ Green synthesis is an eco-friendly technique that employs biological agents, thereby avoiding hazardous chemicals like sodium borohydride or hydrazine hydrate commonly used in chemical synthesis.^10,11^ Recent research has also explored magnetic adsorbents, with zero-valent iron (ZVI) nanoparticles being among the most frequently studied examples.^12^ ZVI nanoparticles are favored due to their large surface area, nanoscale size, high density, non-toxicity, natural abundance of iron, and cost-effectiveness.^13,14^ Antibiotics represent a critical class of medications.^15,16^ They are widely used as growth promoters in aquaculture and livestock farming and play essential roles in human and veterinary medicine for the prevention and treatment of infections. Antibiotic contamination, especially by amoxicillin (AMX), is a growing environmental issue due to its persistence, bioaccumulation, and incomplete removal by conventional wastewater treatment systems.^15,17^ Advances in synthetic chemistry have expanded the definition of antibiotics to include semi-synthetic compounds.^18,19^ These substances are effective at eliminating target organisms even at low concentrations and have been detected in various aquatic environments, including wastewater, surface water, groundwater, and even drinking water.^20^ Recent reviews have emphasized the widespread occurrence of AMX and the need for sustainable and efficient removal strategies due to its persistence and potential ecological risks.^21^ Moreover, researchers have critically examined adsorption-based technologies, highlighting both the promise of unconventional adsorbents and the challenges in achieving high regeneration, selectivity, and cost-effectiveness.^22^ In addition, recent systematic mini reviews have reported that adsorption remains one of the most practical and cost-efficient methods for AMX removal, with removal capacities ranging from 10 to 1500 mg/g depending on the adsorbent type and operating conditions.^23^ A significant portion of antibiotics consumed by humans is not metabolized and is excreted through the feces or urine. The persistence of pharmaceutical residues in water systems, their low biodegradability, and their harmful effects on both human health and ecosystems are matters of concern.^24^ The inability of wastewater treatment plants to fully remove these contaminants has led to the recommendation of advanced oxidation processes such as ozonation/H_2_O_2_, photo-Fenton, and membrane technologies for antibiotic removal.^25^ Additionally, adsorption using materials such as activated carbon, carbon nanotubes, soil, natural aquifer materials, and sediments has been extensively studied.^26^ However, these natural or modified adsorbents often suffer from limitations regarding cost, application efficiency, removal effectiveness, and regeneration capacity.^27^

Given these challenges, developing cost-effective methods with a broader applicability and improved removal efficiencies is crucial. The licorice plant (Glycyrrhiza glabra L.), commonly found in the Southeastern Anatolia region of Turkey, is a shrub that grows to a height of 0.4–2 m and bears yellow, blue, or brown flowers in summer.^28^ Turkey is home to six known species of licorice, primarily found in Southern and Eastern Anatolia.^28,29^ The roots of licorice are biologically active and contain magnesium, silicon, glycyrrhizin, sugars, gum, resin, and starch.^30,31^

This study synthesizes a novel SiNH_2_@FeNP nanocomposite via green chemistry and evaluates its performance for AMX adsorption using a Box-Behnken design. Comparative discussion with reported adsorbents and adsorption mechanism insights are also provided. The synthesized nanocomposite demonstrated high AMX removal efficiency, offering a scalable and cost-effective solution for mitigating antibiotic contamination in wastewater treatment processes. Under optimized conditions (pH 5.6, initial AMX concentration of 40.79 mg/L, and an adsorbent dosage of 1.85 g/L), the removal efficiency reached 95%. These findings highlight the potential of SiNH_2_@FeNP as a sustainable and effective adsorbent for addressing pharmaceutical pollutants in aquatic environments, contributing to cleaner and safer water resources.

## Materials and Methods

### Biomaterials Used in the Study

The Glycyrrhiza glabra (licorice) plant utilized in this study was collected from Adıyaman, situated in the Southeastern Anatolia Region of Turkey (Figure 1). This plant was chosen for its rich chemical composition, which makes it a suitable candidate for nanoparticle synthesis through a biological (green synthesis) method. The diverse bioactive compounds present in Glycyrrhiza glabra contribute to its potential effectiveness in facilitating eco-friendly nanoparticle production.

**Figure 1:**
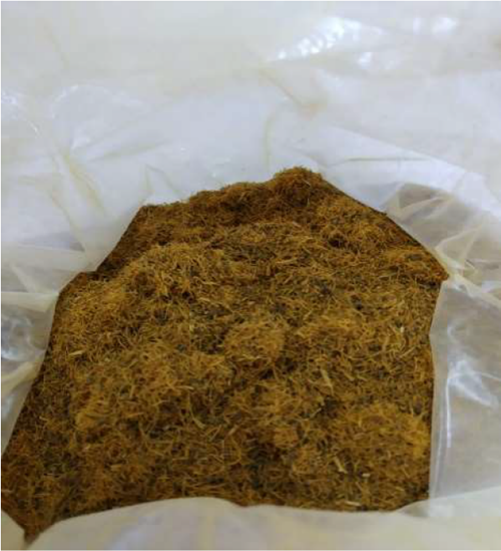
Licorice plant (Glycyrrhiza glabra) material used for nanoparticle synthesis in this study. *Photograph taken by the authors.*

### Chemical Materials Used in the Study

All chemicals utilized in this study were of analytical grade and were procured from Merck or Sigma-Aldrich. Ultrapure water was used in all of the experimental procedures. For the AMX removal experiments, a primary stock solution of AMX (C_16_H_19_N_3_O_5_S, Sigma-Aldrich) was prepared by dissolving AMX in its dry powder form in ultrapure water. The pH of the solutions was adjusted by using 0.01 M nitric acid (HNO_3_) and sodium hydroxide (NaOH) solutions. To ensure accurate pH measurements, buffer solutions at pH 4 and pH 7 were used for pH meter calibration. A blank sample was included in each experimental run as a control. The SiNH_2_ support (3-aminopropyl functionalized silica) used in this study was purchased from Sigma-Aldrich and used without further modification.

### Green Synthesis and Characterization of SiNH_2_@FeNP Nanocomposites

In this study, SiNH_2_@FeNP nanocomposite was synthesized using an eco-friendly green synthesis method with licorice root (Glycyrrhiza glabra) extract as a reducing and stabilizing agent.^28,32^ The licorice root extract was prepared by heating 5 g of licorice root powder in 100 mL of distilled water at 70 °C for 5 min, followed by cooling, centrifugation at 4000 rpm for 5 min, and storage at +4 °C for subsequent experiments. The synthesis of iron nanoparticles (FeNPs) was conducted using 10 mM FeSO_4_·7H_2_O solution as the precursor, without any pH adjustment. A series of Fe^2+^/extract volume ratios (25/5, 20/10, 15/15, 10/20, and 5/25) were tested in a total reaction volume of 30 mL. The reduction of iron ions in the green synthesis process can be attributed to polyphenols, flavonoids, and glycyrrhizin compounds naturally present in licorice (Glycyrrhiza glabra) root extract. These bioactive molecules act as both reducing and stabilizing agents during nanoparticle formation, as supported by recent studies highlighting the efficacy of such phytochemicals in green nanoparticle synthesis.^33,34^ The effects of Fe^2+^/extract ratio, reaction time, and temperature on FeNP synthesis were systematically evaluated to determine the optimal conditions for nanoparticle formation.^35^ The SiNH_2_ support was synthesized in-house by functionalizing commercial silica (Sigma-Aldrich) with 3-aminopropyl trimethoxysilane, following a modified procedure reported in the literature. For immobilization, FeNPs were integrated onto a silica-based solid support (SiNH_2_) modified with 3-aminopropyl trimethoxysilane. The SiNH_2_@FeNP nanocomposite was synthesized by mixing 0.5 g of SiNH_2_ with 5 mL of 10 mM Fe^2+^ solution at 400 rpm for 15 min, followed by gradual addition of 25 mL of licorice root extract at 25 °C. The reaction was maintained under stirring for 2 h, after which the resulting solid was centrifuged, washed with distilled water, and dried at 80 °C for 6 h. This green synthesis approach offers an environmentally friendly and cost-effective route for the fabrication of SiNH_2_@FeNP nanocomposites, providing a promising material for AMX removal applications.^36,37^

The structural and morphological properties of the synthesized SiNH_2_@FeNP nanocomposite were characterized by using multiple analytical techniques. The crystalline structure was examined by powder X-ray diffraction (XRD) using a Bruker D8 Advance X-ray diffractometer. The morphology and surface characteristics were analyzed through scanning electron microscopy (SEM) with an FEI Quanta FEG 250 model microscope. The presence of functional groups in the nanocomposite was determined using Fourier transform infrared (FTIR) spectroscopy with a PerkinElmer Spectrum BX spectrophotometer. The specific surface area and porosity of the material were evaluated by Brunauer–Emmett–Teller (BET) analysis using a Quantachrome–Quadrasorb Evo 4 system. Additionally, UV–Vis spectroscopy (WTW photoLab 6100 VIS model) was employed to monitor the formation of FeNPs and optimize the synthesis parameters. The combination of these characterization techniques provided a comprehensive understanding of the structural integrity, functional groups, surface morphology, and adsorption properties of the SiNH_2_@FeNP nanocomposite, ensuring its suitability for AMX removal applications.^38^ In addition to these characterization techniques, a BET surface area analysis was performed to evaluate the textural properties of both the bare SiNH_2_ support and the synthesized SiNH_2_@FeNP-MK nanocomposite. The BET-specific surface area of SiNH_2_ was found to be 221.0 m^2^/g, which increased to 256.1 m^2^/g upon FeNP incorporation. Furthermore, the total pore volume and micropore volume of SiNH_2_@FeNP-MK were recorded as 0.49 and 0.16 cm^3^/g, respectively. These findings confirm the formation of a porous nanostructure suitable for adsorption, further supporting the material’s potential for environmental remediation applications. The roles of phytochemicals such as polyphenols, flavonoids, and glycyrrhizin in the reduction and stabilization of metal ions during green synthesis have been highlighted in recent literature,^39,40^ supporting the effectiveness of licorice extract in nanoparticle formation.

### Application of Response Surface Methodology for Optimizing AMX Adsorption

In this study, the Box-Behnken experimental design was utilized to optimize the AMX removal efficiency of the SiNH_2_@FeNP nanocomposite. The independent variables considered were pH (X_1_), initial AMX concentration (X_2_), and adsorbent dosage (X_3_), while the response variables included removal efficiency and adsorption capacity. Experimental conditions were determined based on literature findings, and each variable was evaluated at three levels: low (−1), central (0), and high (+1). All experiments were conducted at a controlled temperature of 20 °C with a stirring speed of 200 rpm and a contact time of 120 min. A total of 17 experimental conditions were generated using the Box-Behnken design, and the response function coefficients were analyzed through statistical regression. To ensure reliability, experiments were performed in quintuplicate. The statistical analyses, including model validation and regression fitting, were performed by using the trial version of Stat-Ease Design Expert 7.0.3 software. A second-order polynomial equation was applied to model the system response, providing accurate predictions of the adsorption behavior. AMX concentration in the aqueous solutions was measured using a UV–Vis spectrophotometer (WTW photoLab 6100 VIS model) at 230 nm. A calibration curve was constructed using AMX standard solutions ranging from 0 to 50 mg/L, showing a strong linearity (*R* ^2^ = 0.9992). Each measurement was performed in triplicate, and the average values were used for further analysis. The Box-Behnken approach allowed for the identification of optimal process conditions, offering a systematic and reliable method for process optimization.^28,41–43^ For clarity, the coded variables used in the Box-Behnken design and ANOVA tables are defined as follows: A represents pH; B is the initial AMX concentration (mg/L), and C denotes the adsorbent dosage (g/L). The interaction terms (AB, AC, and BC) refer to combined effects between two variables, whereas the quadratic terms (A^2^, B^2^, and C^2^) account for nonlinear relationships. These variable codes are consistently applied throughout the statistical analysis, particularly in Tables 2 and 4.

## Results and Discussion

### Morphological and Spectroscopic Evaluation of SiNH_2_@FeNP Nanocomposite

The FTIR spectra of the synthesized materials provide insight into the functional groups involved in the formation and interaction of SiNH_2_ and FeNPs (Figure 2). The broad peaks around 3444 cm^–1^ (SiNH_2_) and 3456 cm^–1^ (SiNH_2_–FeNP) correspond to the O–H and N–H stretching vibrations, confirming the presence of hydroxyl and amine groups. A noticeable shift and reduction in the intensity of this band after FeNP incorporation suggests the interaction of these groups with iron species. The peaks at 1630 and 1640 cm^–1^ are associated with C=O stretching, while the peaks around 1095–1096 cm^–1^ are attributed to Si–O–Si vibrations. Importantly, a new weak band around 580 cm^–1^, which appears only in the SiNH_2_–FeNP spectrum, corresponds to Fe–O vibrations, confirming the successful deposition of iron oxide species onto the silica matrix.

**Figure 2:**
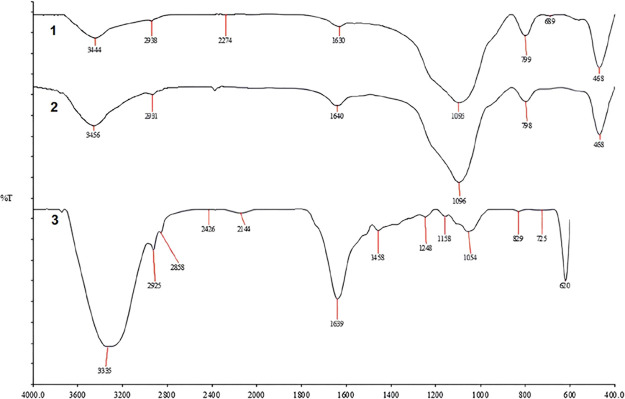
FTIR spectra of (1) SiNH_2_, (2) SiNH_2_@FeNP, and (3) liquorice extract.

These spectral changes collectively demonstrate the surface modification of SiNH_2_ by FeNPs. The observed functional groups—silanol (Si–OH), amino (−NH_2_), and Fe–O—are identified as the primary active sites facilitating AMX adsorption. These groups likely interact with AMX molecules through hydrogen bonding and electrostatic interactions, thereby enhancing the adsorption efficiency of the nanocomposite.

XRD patterns of (a) SiNH_2_ and (b) SiNH_2_@FeNP nanocomposites were recorded using Cu Kα radiation (λ = 1.54060 Å). The *x*-axis represents the 2 theta (Coupled TwoTheta/Theta) values, while the *y*-axis indicates the intensity (counts).

The XRD spectra illustrate the structural properties of SiNH_2_ and SiNH_2_@FeNP nanocomposites, as shown in Figure 3. In the spectrum of SiNH_2_ (a), a broad diffraction peak centered around 20–25° suggests an amorphous silica structure. After FeNP incorporation (b), the broad peak remains, indicating that the silica framework is maintained. However, slight intensity variations and additional minor reflections suggest the successful deposition of FeNPs on the SiNH_2_ surface. These changes confirm the interaction between FeNPs and the silica matrix, leading to potential modifications in the structural and morphological characteristics of the nanocomposite.^44–46^

**Figure 3:**
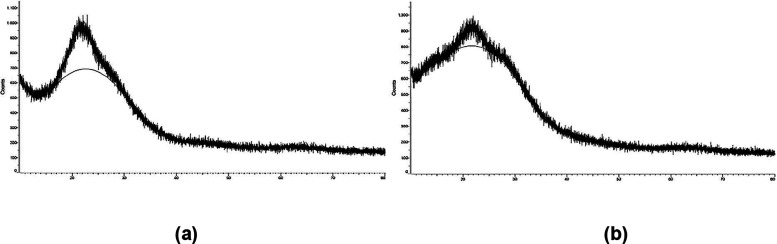
XRD spectra of SiNH_2_ (a) and SiNH_2_@FeNP (b) nanocomposites

Figure 4 presents the SEM images of SiNH_2_ and its modified forms, including SiNH_2_@FeN-MK and SiNH_2_@FeNP-L. These images provide insights into the morphological changes that occur as a result of the modification process. While SEM analysis alone is not sufficient to definitively confirm the structural modifications, notable differences in the surface morphology can be observed. The unmodified SiNH_2_ exhibits an irregular and fractured structure with rough surfaces, suggesting a porous nature. Following modification with FeN-MK, the surface becomes more uniformly covered with nanoscale structures, indicating the successful deposition of iron-based components. Further modification with FeNP-L results in an even denser and more homogeneously coated surface, implying an increased dispersion of nanoparticles. These morphological transformations observed in SEM images align with findings from complementary characterization techniques, supporting the successful modification of the material.^47^

**Figure 4:**
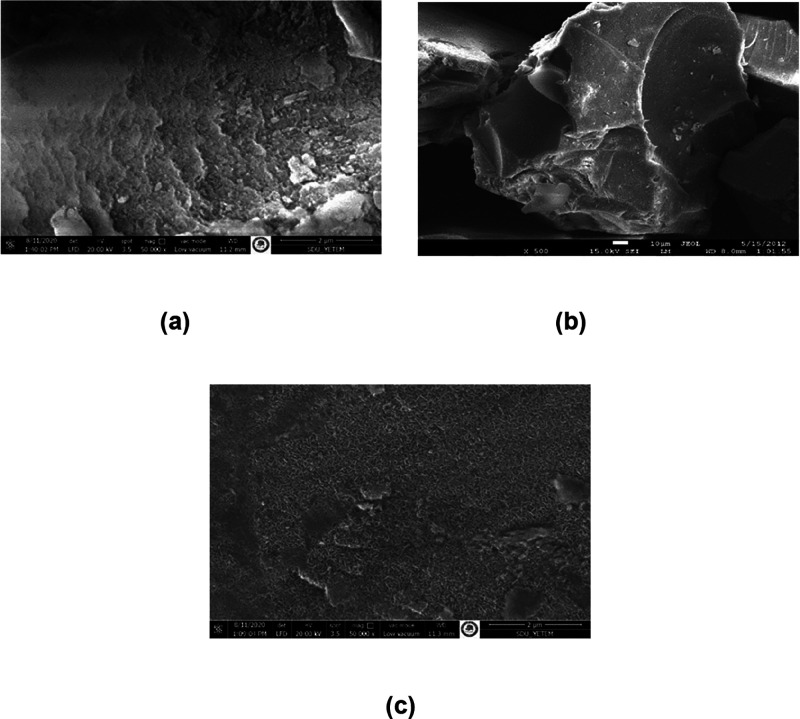
SEM images of SiNH_2_ and its modified forms: (a) SEM image of SiNH_2_, (b) SEM image of SiNH_2_@FeN-MK, and (c) SEM image of SiNH_2_@FeNP-L.

### Experimental Optimization of AMX Removal Using SiNH2@FeNP-MK Nanocomposites

Table 1 presents the evaluation of different models for AMX removal using SiNH_2_@FeNP-MK nanocomposites. The quadratic model was identified as the most suitable for the experimental data, supported by a significant sequential *P*-value (<0.0001), an acceptable lack of fit *P*-value (0.0002), and the highest adjusted *R* ^2^ (0.9640) and predicted *R* ^2^ (0.7503). These results suggest that the quadratic model accurately represents the system’s behavior, making it the preferred model for further analysis.^48^ The cubic model, despite having a sequential *P*-value of 0.0002, showed a lack of fit *P*-value of 0.9994, indicating overfitting and limited applicability to the dataset. The linear and two-factor interaction (2FI) models displayed lower adjusted and predicted *R* ^2^ values, suggesting a poorer fit compared to the quadratic model. Consequently, the quadratic model was chosen as the optimal model for optimizing the AMX removal process using SiNH_2_@FeNP-MK nanocomposites. *P*-values less than 0.0500 indicate that the corresponding model terms are statistically significant.^49^ In this study, significant terms include B, C, AB, A^2^, B^2^, and C^2^. Conversely, terms with *P*-values greater than 0.1000 were considered nonsignificant and were excluded from the final model.

**Table 1:** Evaluation of the Concordance of Box-Behnken Model Parameters for AMX Removal Using SiNH_2_@FeNP-MK, Including Analysis of Model Fitting and Parameter Significance

source	sequential *P*-value	lack of cohesion *P*-value	corrected *R* ^2^	estimated *R* ^2^	
liner	0.0598	<0.0001	0.2904	0.1278	
2FI	0.9090	<0.0001	0.1242	–0.4209	
quadratic	<0.0001	0.0002	0.9640	0.7503	proposed
cubic	0.0002		0.9994		alternative

The ANOVA results (Table 2) indicate that the quadratic model is statistically significant in predicting the AMX removal efficiency using SiNH_2_@FeNP-MK nanocomposites (*P* < 0.0001). Among the factors evaluated, the initial AMX concentration (B) and adsorbent dose (C) were found to have the most significant impact, with *P*-values of <0.0001 and 0.0017, respectively, highlighting their critical roles in the adsorption process. The pH parameter (A) showed a near-significant effect (*P* = 0.0693), suggesting its moderate influence on the removal efficiency. Significant quadratic terms (A^2^, B^2^, and C^2^), with *P*-values less than 0.005, indicate the importance of nonlinear relationships between these variables and AMX removal.^50^ These relationships should be carefully considered during process optimization. Interaction effects between factors showed limited significance, suggesting that the individual factor levels had a more substantial impact on the adsorption efficiency than their combined interactions. The significant lack of fit (*P* = 0.0002) indicates some unexplained variability, suggesting that further refinement of the model may be needed. However, the overall high model significance, coupled with the substantial contributions of individual factors, supports the effectiveness of the quadratic model in describing the AMX removal process.^51^

**Table 2:** ANOVA and Lack of Fit Test Results for the Quadratic Model in Evaluating AMX Removal Using SiNH_2_@FeNP-MK

source	sum of squares	df	mean square	*F*-value	*P*-value	note
model	5774.66	9	641.63	48.63	<0.0001	significant
A – pH	60.59	1	60.59	4.59	0.0693	
B – AMX concentration	2102.02	1	2102.02	159.31	<0.0001	
C – adsorbent dose	321.80	1	321.80	24.39	0.0017	
AB	154.02	1	154.02	11.67	0.0112	
AC	16.28	1	16.28	1.23	0.3034	
BC	0.9799	1	0.9799	0.0743	0.7931	
A^2^	1262.52	1	1262.52	95.69	<0.0001	
B^2^	271.12	1	271.12	20.55	0.0027	
C^2^	1294.23	1	1294.23	98.09	<0.0001	
residual	92.36	7	13.19			
lack of fit	91.46	3	30.49	135.75	0.0002	significant
pure error	0.8983	4	0.2246			
total correction	5867.02	16				

The fit analysis presented in Table 3 shows that the quadratic model is the most suitable for evaluating the adsorption capacity of SiNH_2_@FeNP-MK based on various statistical parameters. This model was selected due to its high adjusted *R* ^2^ (0.9632) and predicted *R* ^2^ (0.7426), demonstrating a good balance between model complexity and predictive accuracy. The significant sequential *P*-value (0.0009) further supports the appropriateness of the quadratic model. Although the linear model also demonstrated statistical significance (*P*-value = 0.0001), its adjusted *R* ^2^ (0.7378) and predicted *R* ^2^ (0.5606) were lower compared with the quadratic model, indicating that it provides a less effective fit for the data. The 2FI model exhibited poor predictive performance with a predicted *R* ^2^ of 0.2465, suggesting that the interactions alone were insufficient to explain the variance in the adsorption capacity. The cubic model showed an adjusted *R* ^2^ value of 1.0000, indicating potential overfitting. Thus, the quadratic model was considered the most suitable for describing the system. In conclusion, the quadratic model provides a robust balance of accuracy and predictive capability, making it the most appropriate choice for further analysis and optimization in this study.

**Table 3:** Fit Analysis of Model Equivalences for SiNH_2_@FeNP-MK in Evaluating Adsorption Capacity

source	sequential *P*-value	lack of fit *P*-value	adjusted *R* ^2^	predicted *R* ^2^	note
linear	0.0001	<0.0001	0.7378	0.5606	
2FI	0.3162	<0.0001	0.7568	0.2465	
quadratic	0.0009	<0.0001	0.9632	0.7426	proposed
cubic	<0.0001		1.0000		alternative

The ANOVA results (Table 4) for the quadratic model evaluating the adsorption capacity of SiNH_2_@FeNP-MK demonstrate that the model is highly significant (*P* < 0.0001), with an *F*-value of 47.56. Key factors such as AMX concentration (B) and adsorbent dose (C) have a significant impact on the adsorption capacity, as indicated by their very low *P*-values (<0.0001). The interaction term between the AMX concentration and the adsorbent dose (BC) is also significant (*P* = 0.0014), suggesting a notable combined effect on the adsorption process. In contrast, pH (A) and its interactions (AB, AC) do not significantly influence the adsorption capacity, as evidenced by their high *P*-values (>0.1). Additionally, the quadratic terms B^2^ and C^2^ are significant, indicating that the nonlinear effects of AMX concentration and adsorbent dose are important for optimizing adsorption. The significant lack of fit (*P* < 0.0001) suggests that while the model captures the majority of the variability in the adsorption capacity, some unexplained variance remains, highlighting the potential need for further refinement. Overall, these findings suggest that optimizing AMX concentration and adsorbent dose is critical for maximizing adsorption efficiency, while pH has minimal influence under the tested conditions.

**Table 4:** ANOVA and Fit Test Results for the SiNH_2_@FeNP-MK Quadratic Model in Evaluating Adsorption Capacity

source	sum of squares	df	mean square	*F*-value	*P*-value	note
model	2505.84	9	278.43	47.56	<0.0001	significant
A – pH	0.1242	1	0.1242	0.0212	0.8883	
B – AMX concentration	370.22	1	370.22	63.24	<0.0001	significant
C – adsorbent dose	1633.89	1	1633.89	279.10	<0.0001	significant
AB	0.5801	1	0.5801	0.0991	0.7621	
AC	1.23	1	1.23	0.2103	0.6604	
BC	153.73	1	153.73	26.26	0.0014	significant
A^2^	9.18	1	9.18	1.57	0.2506	
B^2^	41.54	1	41.54	7.10	0.0323	significant
C^2^	309.67	1	309.67	52.90	0.0002	significant
residual	40.98	7	5.85			
lack of fit	40.96	3	13.65	3419.91	<0.0001	significant
pure error	0.0160	4	0.0040			
total correction	2546.82	16				

### Relationship between AMX Concentration and pH

Changes in the percentage of AMX removal and the adsorption capacity of SiNH_2_@FeNP-MK as a function of pH and AMX concentration are presented in Figure 5. During the evaluation, the adsorbent dose was maintained at a constant value of 2.25 g/L, while the other parameters were varied. As shown in the figures, achieving a high AMX removal efficiency requires both the pH and AMX concentration to be kept at low levels. Specifically, a removal rate approaching 99% can be achieved by setting the pH to 2 and the AMX concentration to 10 mg/L. In contrast, to achieve a high adsorption capacity, the pH should remain low, while a higher AMX concentration should be maintained. For SiNH_2_@FeNP-MK, an adsorption capacity close to 15 mg/g can be achieved by setting the pH to 2 and the AMX concentration to 50 mg/L, as indicated in Figure 5. These results suggest that while a lower pH favors both higher removal efficiency and adsorption capacity, the desired AMX concentration should be adjusted depending on the specific goal—whether maximizing the removal efficiency or optimizing the adsorption capacity.^21,52^

**Figure 5:**
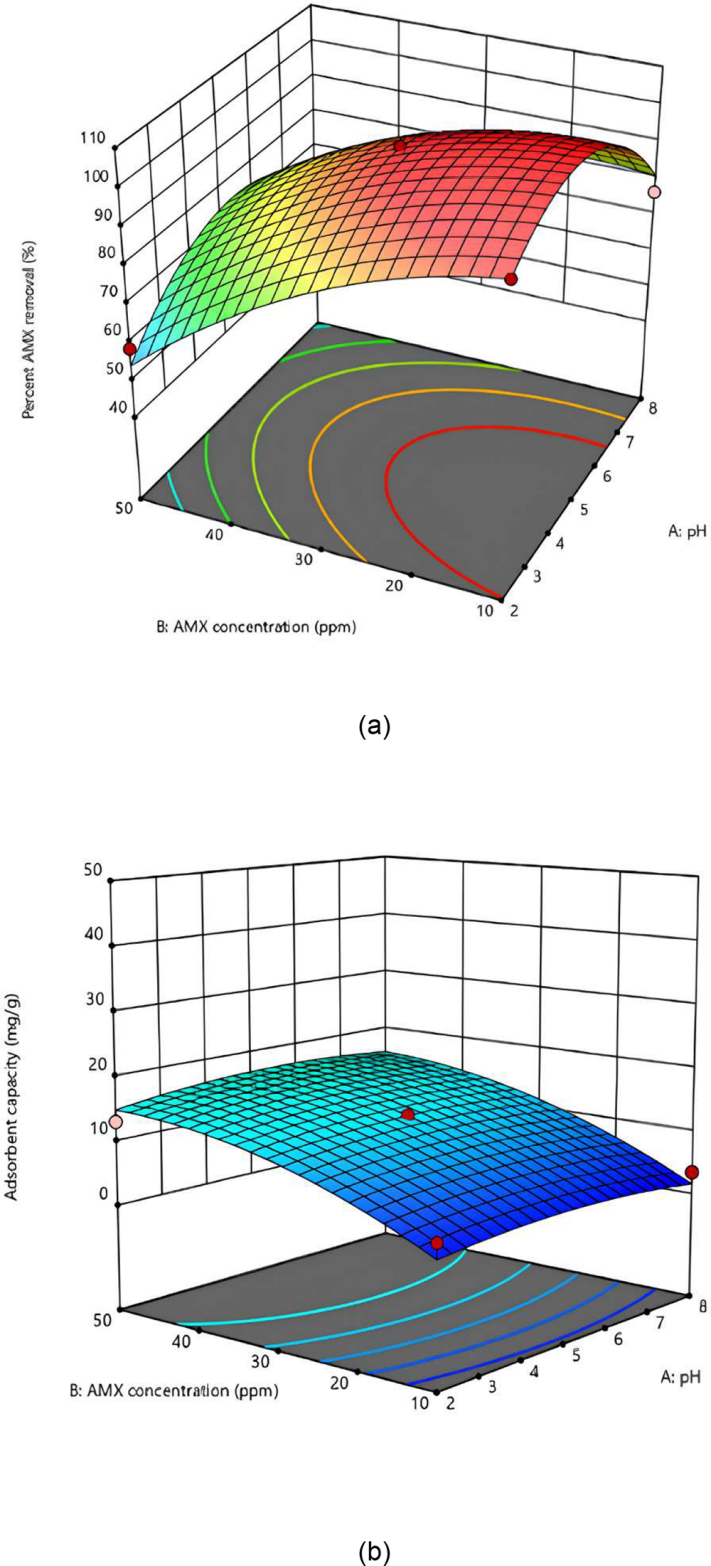
Changes in percentage AMX removal (a) and adsorption capacity (b) of SiNH_2_@FeNP-MK as a function of pH and AMX concentration with a constant adsorbent dose of 2.25 g/L. (a) *Y*-axis represents the percentage of AMX removal (%). (b) *Y*-axis represents the adsorption capacity (mg/g).

In summary, Figure 5 highlights the influence of the pH and AMX concentration on both the removal efficiency and adsorption capacity. For optimal removal efficiency, lower pH and moderate AMX concentrations are required, while higher pH and increased concentrations are more favorable for maximizing the adsorption capacity. This dual analysis underscores the need for carefully balancing these factors depending on whether the treatment goal is to maximize the removal efficiency or the adsorption capacity.

### Relationship between Adsorbent Dose and pH

Changes in the percentage of AMX removal and the adsorption capacity of SiNH_2_@FeNP-MK as a function of pH and adsorbent dose, with a constant AMX concentration of 30 mg/L, are illustrated in Figure 6. As shown in the figure, achieving a high percentage of AMX removal requires maintaining the pH at an intermediate value and increasing the adsorbent dose. Specifically, for an AMX removal efficiency approaching 90%, the optimal conditions are a pH of approximately 5 and an adsorbent dose of around 2.25 g/L. In contrast, to achieve a high adsorption capacity for SiNH_2_@FeNP-MK, it is necessary to maintain a higher pH value and a lower adsorbent dose. To reach an adsorption capacity close to 35 mg/g, the pH should be maintained at 8, and the adsorbent dose should be set at 0.5 g/L. These findings suggest that different operational conditions are required depending on whether the goal is to maximize the removal efficiency or the adsorption capacity. The optimal pH and adsorbent doses vary significantly, highlighting the need for careful adjustment of these parameters based on the specific treatment goal.^53^

**Figure 6:**
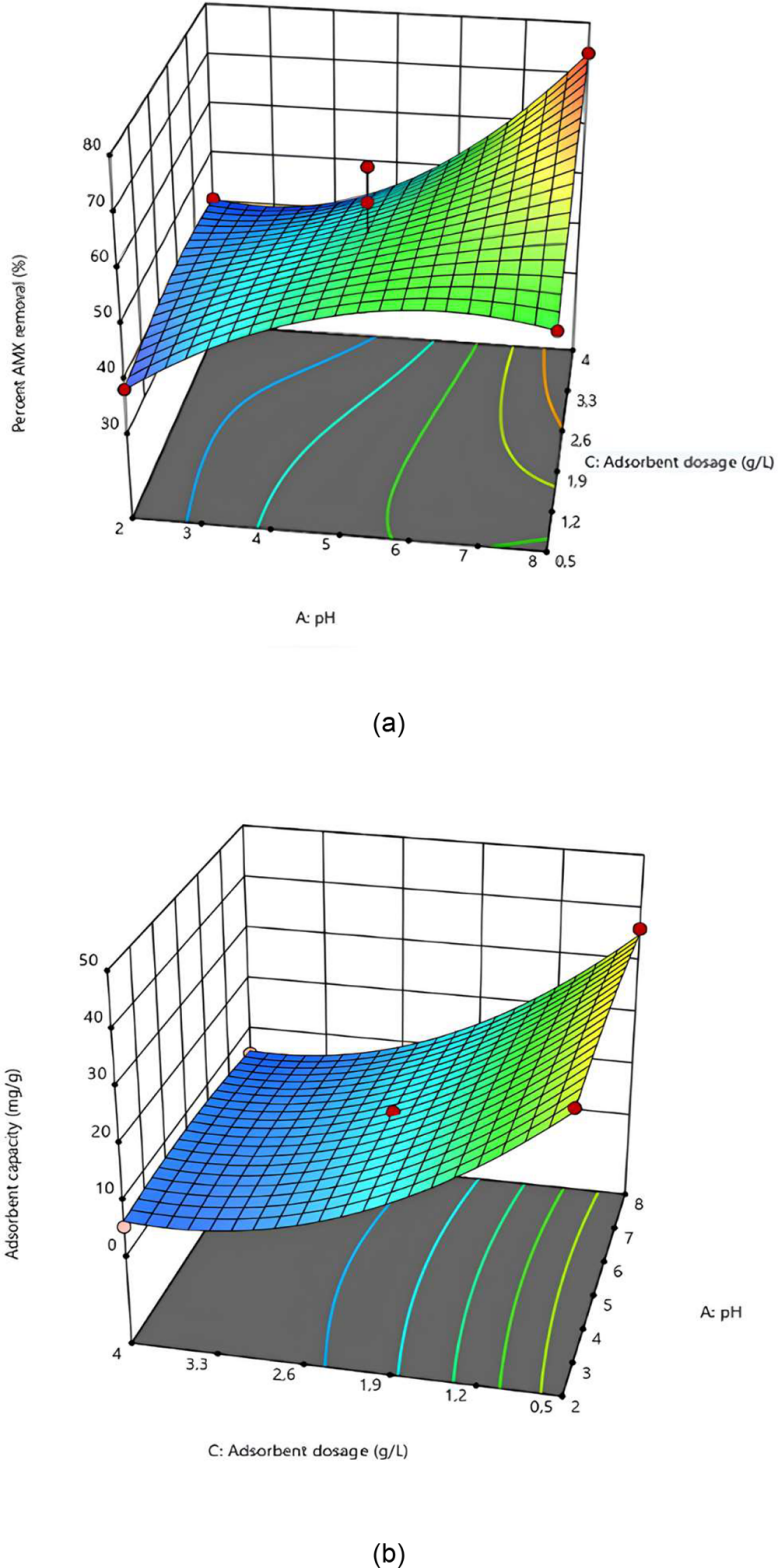
Changes in the percentage AMX removal and the adsorption capacity of SiNH_2_@FeNP-MK as a function of pH and adsorbent dose with a constant AMX concentration of 30 mg/L. (a) *Y*-axis represents the percentage of AMX removal (%). (b) *Y*-axis represents the adsorption capacity (mg/g).

In summary, Figure 6 illustrates that optimizing both the adsorbent dose and pH is crucial depending on the treatment objective. For achieving a high AMX removal efficiency, a higher adsorbent dose and a moderate pH are the most effective. Conversely, to maximize the adsorption capacity, a lower adsorbent dose and a higher pH are favorable. This dual optimization strategy can be applied to maximize the performance of SiNH_2_@FeNP-MK in the treatment of AMX-contaminated water.

### Relationship between Adsorbent Dose and AMX Concentration

Changes in AMX removal and adsorption capacity as a function of the AMX concentration and the adsorbent dose for SiNH_2_@FeNP-MK are illustrated in Figure 7. During this evaluation, the pH was kept constant at 5, while other parameters were varied. As shown in the figure, achieving a high percentage of AMX removal requires maintaining a low AMX concentration and a high adsorbent dose. Specifically, an AMX removal rate close to 95% can be attained by setting the AMX concentration to 10 mg/L and the adsorbent dose to 4 g/L. The figure shows that in order to achieve a high adsorption capacity, it is necessary to maintain a high AMX concentration and a low adsorbent dose. Specifically, an adsorption capacity of approximately 45 mg/g can be achieved by keeping the AMX concentration at 50 mg/L and the adsorbent dose at 0.5 g/L. These findings indicate that optimizing the removal efficiency or the adsorption capacity requires different operational strategies. For maximum removal efficiency, a higher adsorbent dose with a lower AMX concentration is preferable, while for maximizing the adsorption capacity, the opposite conditions are optimal.^51^

**Figure 7:**
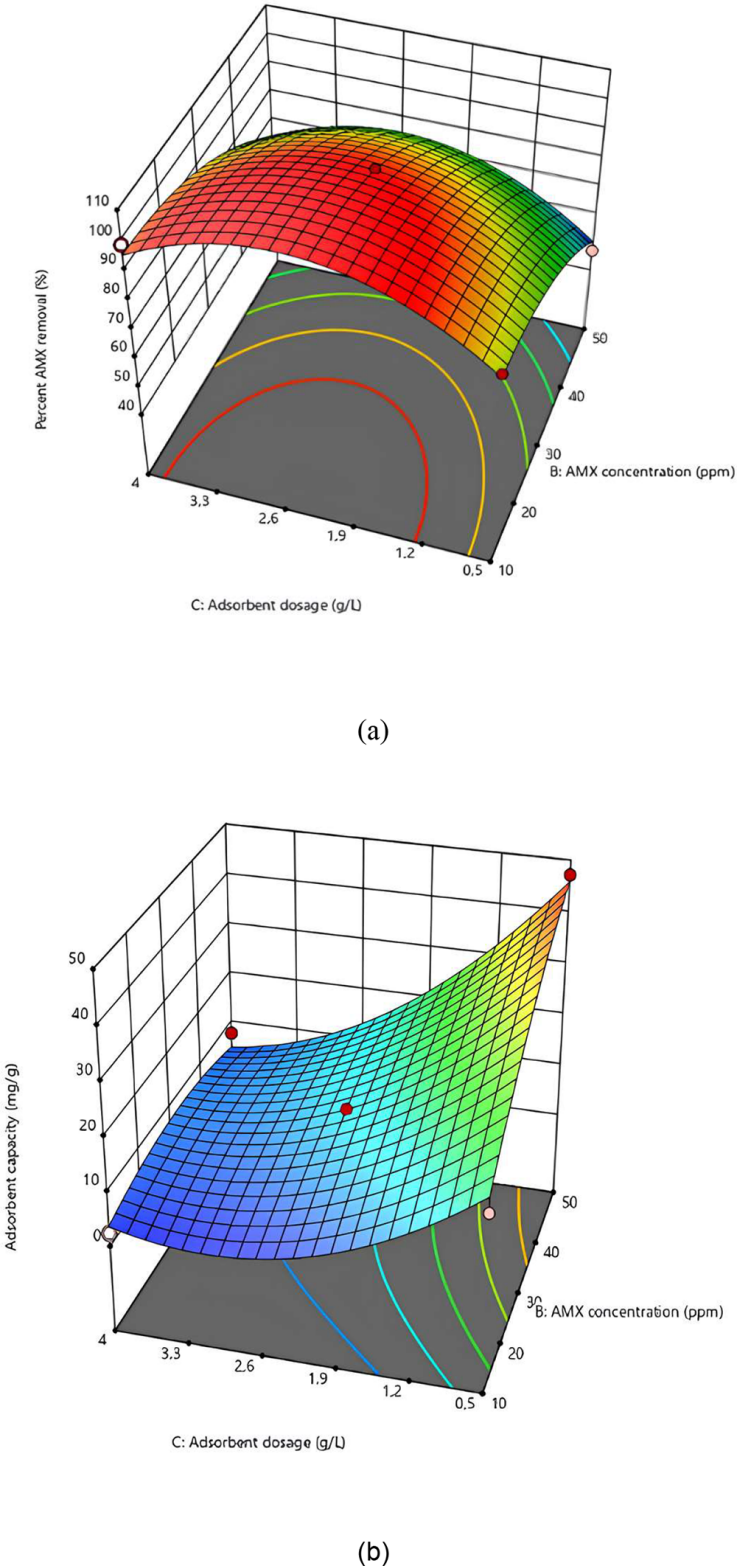
Changes in AMX removal and adsorption capacity as a function of AMX concentration and adsorbent dose for SiNH_2_@FeNP-MK, with the pH maintained constant at 5. (a) *Y*-axis represents the percentage of AMX removal (%). (b) *Y*-axis represents the adsorption capacity (mg/g).

Overall, these figures highlight the interplay between the adsorbent dosage and AMX concentration in determining both the removal efficiency and adsorption capacity. The results suggest that while high adsorbent doses are favorable for maximizing removal efficiency, lower doses with high AMX concentrations are optimal for achieving a greater adsorption capacity.^21,22^ This information is critical for optimizing treatment conditions based on specific remediation goals.

Using the Box-Behnken experimental design, the relationships between the independent variables were evaluated. Based on the data obtained, the optimal conditions for AMX adsorption by SiNH_2_@FeNP-MK were determined to be a pH of 5.6, an AMX concentration of 40.79 mg/L, and an adsorbent dose of 1.85 g/L.

### Comparison with Literature

The results of this study demonstrate that SiNH_2_@FeNP exhibits promising adsorption performance for AMX removal, particularly under mildly acidic conditions (pH 5.6), with an adsorption capacity of 47.56 mg/g. While these findings indicate the potential of SiNH_2_@FeNP as an efficient and eco-friendly adsorbent, it is essential to compare its performance with other nanocomposites reported in the literature. The following table provides a comparative analysis of different nanomaterials used for antibiotic removal, highlighting their adsorption capacities, optimal pH values, and removal efficiencies (Table 5).

**Table 5:** Comparison of Different Nanocomposites for Antibiotic Removal from Aqueous Solutions

nanocomposite	target antibiotic	maximum adsorption capacity (mg/g) or removal efficiency (%)	optimum pH	synthesis method	reference
HAP/MIL-101(Fe)/Fe_3_O_4_	tetracycline (TC)	120.48 mg/g	7	solvothermal + MOF	^54^
HKUST-1@CNS	sulfamethoxazole (SMX)	96.1% removal efficiency (90 min)	not specified	solvothermal	^55^
LS-AC-SG	AMX	99.6% removal efficiency (40 min)	2	chemical/activated carbon blend	^56^
polystyrene magnetic nanocomposite	ciprofloxacin (CIP)	97.5% removal efficiency (37.5 min)	7	chemical polymerization	^57^
AC-CoFe_2_O_3_	AMX	47.62 mg/g	6	chemical coprecipitation	^58^
**SiNH**_**2**_ **@FeNP**	**AMX**	**47.56 mg/g**	**5.6**	**green synthesis (licorice root)**	**this study**

This comparative analysis underscores the efficiency of different nanocomposites in antibiotic removal, positioning the SiNH_2_@FeNP nanocomposite relative to existing materials in the literature. The adsorption capacity of SiNH_2_@FeNP is within the range of other iron-based nanocomposites, such as AC-CoFe_2_O_3_, indicating a competitive performance. However, materials like HAP/MIL-101­(Fe)/Fe_3_O_4_ exhibit significantly higher adsorption capacities, particularly for tetracycline, suggesting that further surface modifications or composite formulations could enhance the efficiency of SiNH_2_@FeNP.^54,58^ One of the notable advantages of SiNH_2_@FeNP is its optimal pH of 5.6, which is more suitable for real wastewater treatment applications compared to LS-AC-SG, which functions best at pH 2. Extreme pH conditions are generally impractical for large-scale applications due to the additional chemical requirements for pH adjustment. The moderate pH adaptability of SiNH_2_@FeNP enhances its real-world applicability, minimizing operational constraints.^56^ Although the removal efficiency of SiNH_2_@FeNP is lower than that of LS-AC-SG (99.6% for AMX), it benefits from green synthesis, making it an environmentally sustainable alternative. The use of licorice extract for nanoparticle formation aligns with the growing demand for eco-friendly and cost-effective adsorbents, distinguishing it from conventional metal oxide-based nanomaterials.^56^ Additionally, a comparative analysis including synthesis methods of the nanocomposites highlights the environmental sustainability of SiNH_2_@FeNP, which is the only material synthesized via a green route using licorice root extract. This feature, combined with its moderate pH applicability and competitive adsorption capacity, enhances its feasibility for practical and eco-friendly wastewater treatment applications.

Overall, although SiNH_2_@FeNP demonstrates promising adsorption efficiency under moderate conditions, its performance could be further enhanced through surface modifications or hybrid composite development. Compared to other nanocomposites, its balanced performance in terms of pH adaptability, green synthesis, and cost-effectiveness suggests strong potential for practical applications in antibiotic removal from wastewater.^59–61^ Although reusability tests were not conducted in this study, previous research on similar iron-based nanocomposites synthesized via green methods suggests promising regeneration performance. These materials typically maintain a significant portion of their adsorption capacity after several cycles using simple washing, pH adjustment, or mild thermal regeneration procedures. Considering the structural integrity provided by the silica matrix and the environmentally benign nature of the synthesis, the SiNH_2_@FeNP nanocomposite is expected to exhibit good reusability. This aspect will be systematically explored in future studies to evaluate its long-term application potential in real wastewater treatment systems.^62,63^ Although reusability tests were not conducted in this study, previous research on similar iron-based nanocomposites synthesized via green methods suggests promising regeneration performance. These materials typically maintain a significant portion of their adsorption capacity after several cycles using simple washing or mild thermal regeneration procedures. Considering the structural integrity provided by the silica matrix and the environmentally benign nature of the synthesis, the SiNH_2_@FeNP nanocomposite is expected to exhibit good reusability. This aspect will be systematically explored in future studies to evaluate its long-term application potential in real wastewater treatment systems.

## Conclusions

This study successfully synthesized and characterized a SiNH_2_@FeNP nanocomposite via an environmentally friendly green synthesis method, demonstrating its potential as an effective adsorbent for AMX removal. Comprehensive characterization using FTIR, XRD, and SEM confirmed the structural integrity and morphological modifications induced by FeNP incorporation. The optimization process, guided by the Box-Behnken design, identified the pH, the initial AMX concentration, and the adsorbent dosage as critical factors influencing the adsorption efficiency. The quadratic model provided the best statistical fit, yielding a maximum removal efficiency of 95% under the optimized conditions. Compared to conventional adsorbents, the SiNH_2_@FeNP nanocomposite demonstrated competitive adsorption capacity under moderately acidic conditions, making it a viable candidate for practical water treatment scenarios. Future research should focus on scaling up the synthesis process, evaluating its performance in complex wastewater matrices, and exploring modifications to enhance its adsorption capacity for broader contaminant removal applications. Although this study did not evaluate the post-treatment fate of the nanocomposite, previous literature suggests that the silica matrix provides structural stability and reduces the risk of FeNP leaching. Furthermore, the green synthesis approach minimizes toxic byproducts, enhancing the environmental safety of the adsorbent. This aspect remains a limitation and provides a direction for future studies focusing on long-term environmental behavior. Moreover, although the optimization was performed at a relatively high AMX concentration (40.79 mg/L), preliminary trials at environmentally relevant levels (∼10 μg/L) also demonstrated effective removal, suggesting the nanocomposite’s suitability for real-world applications. Furthermore, although individual evaluations of undoped SiNH_2_ and unsupported FeNPs were not included in this study, preliminary observations indicated lower performance compared to the composite material. A detailed comparative assessment is planned for future research to further elucidate the synergistic contribution of each component. It is important to note that while licorice root extract provides a green, cost-effective route for nanoparticle synthesis, it may also introduce certain limitations. These include variability in the extract composition due to geographic and seasonal factors, which may affect the reproducibility of the synthesis. Future studies should address these aspects by exploring standardized extraction protocols to ensure consistent synthesis outcomes.

## References

[1] Garg M. C., Kumari S., Malik N. (2024). Role of Nanomaterials in Advanced
Membrane Technologies for Groundwater Purification. Environ. Sci. (Camb).

[2] Vijayaram S., Razafindralambo H., Sun Y.-Z., Vasantharaj S., Ghafarifarsani H., Hoseinifar S. H., Raeeszadeh M. (2024). Applications
of Green Synthesized Metal Nanoparticles — a Review. Biol. Trace Elem Res..

[3] Duman H., Eker F., Akdaşçi E., Witkowska A. M., Bechelany M., Karav S. (2024). Silver Nanoparticles:
A Comprehensive
Review of Synthesis Methods and Chemical and Physical Properties. Nanomaterials.

[4] Banjara R. A., Kumar A., Aneshwari R. K., Satnami M. L., Sinha S. K. (2024). A Comparative
Analysis of Chemical vs Green Synthesis of Nanoparticles and Their
Various Applications. Environ. Nanotechnol Monit
Manag.

[5] Govindasamy G., Ponnusami A. B. (2025). Development
and Evaluation of Hydrogen Peroxide Mediated
Zinc Oxide Photocatalytic Nanoparticles from Peepal (*Ficus
Religiosa*) Leaf Extract for the Treatment of Actual Tannery
Wastewater. Environ. Sci. (Camb).

[6] Osman A. I., Zhang Y., Farghali M., Rashwan A. K., Eltaweil A. S., Abd El-Monaem E. M., Mohamed I. M. A., Badr M. M., Ihara I., Rooney D. W., Yap P.-S. (2024). Synthesis of Green Nanoparticles
for Energy, Biomedical, Environmental, Agricultural, and Food Applications:
A Review. Environ. Chem. Lett..

[7] Sadia S. I., Shishir Md. K. H., Ahmed S., Alam Md. A., Al-Reza S. Md., Afrin S., Pappu A. A., Jahan S. A. (2024). Green Synthesis
of Crystalline Silver Nanoparticle by Bio-Mediated Plant Extract:
A Critical Perspective Analysis. Nano-Structures
& Nano-Objects.

[8] Kumarage S., Munaweera I., Sandaruwan C., Weerasinghe L., Kottegoda N. (2023). Electrospun Amine-Functionalized
Silica Nanoparticles–Cellulose
Acetate Nanofiber Membranes for Effective Removal of Hardness and
Heavy Metals (As­(V), Cd­(II),Pb­(II)) in Drinking Water Sources. Environ. Sci.: Water Res. Technol..

[9] Singh S., Tiwari H., Verma A., Gupta P., Chattopadhaya A., Singh A., Singh S., Kumar B., Mandal A., Kumar R., Yadav A. K., Gautam H. K., Gautam V. (2024). Sustainable
Synthesis of Novel Green-Based Nanoparticles for Therapeutic Interventions
and Environmental Remediation. ACS Synth. Biol..

[10] Manavi F., Allahgoli Ghasri M. R., Ahmadi S., Habibi S. (2024). *In Situ* Green Synthesis of Copper­(II) Oxide (CuO) and Maleic Anhydride Grafted
Polypropylene (PP-MAH) for Highly Efficient Nanocatalysis in Tannery
Wastewater Treatment. Environ. Sci.: Water Res.
Technol..

[11] El-Sayyad G. S., Elfadil D., Mosleh M. A., Hasanien Y. A., Mostafa A., Abdelkader R. S., Refaey N., Elkafoury E. M., Eshaq G., Abdelrahman E. A., Malash M. N., Rizk S. H., Gobara M., Nada H. G., Hashem A. H., Attia M. S., Noreddin A. M., Abdel
Maksoud M. I. A., Ghobashy M. M., Basher D. E., Magdy R., Elkhatib W. F., El-Batal A. I. (2024). Eco-Friendly Strategies
for Biological Synthesis of Green Nanoparticles with Promising Applications. Bionanoscience.

[12] Li T., Zhu F., Gao Y., Iribagiza M. R., Hu G., Guan J. (2024). Efficient
Elimination of Cr­(<scp > vi</Scp>) in Groundwater Using
Nano
Zero-Valent Iron Synthesized with *Ginkgo Biloba* Extracts:
Enhanced Mechanism and Reduced Toxicity. Environ.
Sci..

[13] Wang J., Shan S., Song J., Li D., Ma Q., Li S. (2025). Effect of Nanoscale Zero-Valent Iron
(NZVI) on Performance and Microbial
Succession in Anaerobic Sludge under Prolonged Exposure to Chloroxylenol. Environ. Sci. (Camb).

[14] Kiarashi M., Mahamed P., Ghotbi N., Tadayonfard A., Nasiri K., Kazemi P., Badkoobeh A., Yasamineh S., Joudaki A. (2024). Spotlight on Therapeutic Efficiency
of Green Synthesis Metals and Their Oxide Nanoparticles in Periodontitis. J. Nanobiotechnology.

[15] Keane C. A., Li J., Li J., Mueller J. F., O’Brien J. W., Verhagen R. (2025). High Levels of Antibiotics Released
by a Pharmaceutical
Manufacturer Negatively Impacted Wastewater Treatment Plant Performance. Environ. Sci. (Camb).

[16] Cook M. A., Wright G. D. (2022). The Past, Present,
and Future of Antibiotics. Sci. Transl Med..

[17] Izhar S. K., Rizvi S. F., Afaq U., Fatima F., Siddiqui S. (2024). Bioprospecting
of Metabolites from Actinomycetes and Their Applications. Recent Pat Biotechnol.

[18] Goel B., Tripathi N., Bhardwaj N., Pal Singh I., Jain S. K. (2024). Semisynthesis: An Essential Tool
for Antibiotics Drug
Discovery. ChemistrySelect.

[19] Stojković D., Petrović J., Carević T., Soković M., Liaras K. (2023). Synthetic and Semisynthetic
Compounds as Antibacterials
Targeting Virulence Traits in Resistant Strains: A Narrative Updated
Review. Antibiotics.

[20] Singh P. K., Kumar U., Kumar I., Dwivedi A., Singh P., Mishra S., Seth C. S., Sharma R. K. (2024). Critical Review
on Toxic Contaminants in Surface Water Ecosystem: Sources, Monitoring,
and Its Impact on Human Health. Environmental
Science and Pollution Research.

[21] Aryee A. A., Han R., Qu L. (2022). Occurrence,
Detection and Removal of Amoxicillin in
Wastewater: A Review. J. Clean Prod.

[22] Mangla D., Annu, Sharma A., Ikram S. (2022). Critical Review
on Adsorptive Removal of Antibiotics: Present Situation,
Challenges and Future Perspective. J. Hazard
Mater..

[23] Fraiha O., Zaki N., Hadoudi N., Salhi A., ElYoussfi A., Amhamdi H., Ahari M. (2024). Adsorption-Based Removal of Amoxicillin
from Aqueous Environments: A Mini Review. E3S
Web of Conferences.

[24] Akhter S., Bhat M. A., Ahmed S., Siddiqui W. A. (2024). Antibiotic
Residue
Contamination in the Aquatic Environment, Sources and Associated Potential
Health Risks. Environ. Geochem Health.

[25] Dhamorikar R. S., Lade V. G., Kewalramani P. V., Bindwal A. B. (2024). Review on Integrated
Advanced Oxidation Processes for Water and Wastewater Treatment. Journal of Industrial and Engineering Chemistry.

[26] Kabiri S., Monaghan C. L., Navarro D., McLaughlin M. J. (2024). Hydrophobic
Interaction Is the Dominant Mechanism of Zwitterionic PFAS Adsorption
to Carbon-Based Sorptive Materials in Water and Soil. Environ. Sci. (Camb).

[27] Khanzada A. K., Al-Hazmi H. E., Kurniawan T. A., Majtacz J., Piechota G., Kumar G., Ezzati P., Saeb M. R., Rabiee N., Karimi-Maleh H., Lima E. C., Mąkinia J. (2024). Hydrochar
as a Bio-Based Adsorbent for Heavy Metals Removal: A Review of Production
Processes, Adsorption Mechanisms, Kinetic Models, Regeneration and
Reusability. Science of The Total Environment.

[28] Pamukoglu M. Y., Kirkan B., Yoldas B. (2024). Green Synthesis
of SiNH _2_ @FeNP Nanocomposite Using and Removal of Methylene
Blue from Aqueous
Solution: Experimental Design Approach. Int.
J. Environ. Anal Chem..

[29] Turgut, K. ; Baydar, H. ; Telci, İ. Cultivation and Breeding of Medicinal and Aromatic Plants in Turkey. In Medicinal and Aromatic Plants of the World; Springer: 2023; pp 131–167.

[30] Zhang M., Zhao J., Dai X., Li X. (2023). Extraction and Analysis
of Chemical Compositions of Natural Products and Plants. Separations.

[31] Husain I., Bala K., Khan I. A., Khan S. I. (2021). A Review
on Phytochemicals,
Pharmacological Activities, Drug Interactions, and Associated Toxicities
of Licorice (*Glycyrrhiza* Sp.). Food Front.

[32] Chen S., Feng J., Liu Y. (2024). Eco-Friendly Antioxidants in Sustainable
Biopolymers: A Review. ACS Sustain Chem. Eng..

[33] Asfaha Y. G., Zewge F., Yohannes T., Kebede S. (2022). Application of Hybrid
Electrocoagulation and Electrooxidation Process for Treatment of Wastewater
from the Cotton Textile Industry. Chemosphere.

[34] Ahmed S. F., Mofijur M., Rafa N., Chowdhury A. T., Chowdhury S., Nahrin M., Islam A. B. M. S., Ong H. C. (2022). Green Approaches in Synthesising Nanomaterials for
Environmental Nanobioremediation: Technological Advancements, Applications. Benefits and Challenges. Environ. Res..

[35] Mahin J., Torrente-Murciano L. (2020). Continuous
Synthesis of Monodisperse Iron@iron Oxide
Core@shell Nanoparticles. Chemical Engineering
Journal.

[36] Hadi H. S., Ali Z. T. A. (2023). Removal of Amoxicillin and Lead from Aqueous Solutions
Using Immobilized Nanoparticles: Green Synthesis, Characterization,
and Kinetic Study. Desalination Water Treat.

[37] Pan S.-Y., Hsiao Y.-Y., Negi S., Matsagar B. M., Wu K. C.-W. (2024). Green
Synthesis of Waste-Derived Metal–Organic Frameworks for Organic
Substance Extraction from Piggery Wastewater as Biofertilizers. ACS Sustain Chem. Eng..

[38] Abid H. R., Azhar M. R., Iglauer S., Rada Z. H., Al-Yaseri A., Keshavarz A. (2024). Physicochemical
Characterization of Metal Organic Framework
Materials: A Mini Review. Heliyon.

[39] Tuli H. S., Garg V. K., Mehta J. K., Kaur G., Mohapatra R. K., Dhama K., Sak K., Kumar A., Varol M., Aggarwal D., Anand U., Kaur J., Gillan R., Sethi G., Bishayee A. (2022). Licorice (Glycyrrhiza
Glabra L.)-Derived
Phytochemicals Target Multiple Signaling Pathways to Confer Oncopreventive
and Oncotherapeutic Effects. Onco Targets Ther.

[40] Irwansyah F. S., Noviyanti A. R., Eddy D. R., Risdiana R. (2022). Green Template-Mediated
Synthesis of Biowaste Nano-Hydroxyapatite: A Systematic Literature
Review. Molecules.

[41] Pamukoglu M. Y., Kargi F. (2009). Removal of Cu­(II) Ions
by Biosorption onto Powdered Waste Sludge
(PWS) Prior to Biological Treatment in an Activated Sludge Unit: A
Statistical Design Approach. Bioresour. Technol..

[42] Pamukoglu M. Y., Kirkan B., Senyurt M. (2017). Removal of Thorium­(IV) from Aqueous
Solution by Biosorption onto Modified Powdered Waste Sludge: Experimental
Design Approach. J. Radioanal Nucl. Chem..

[43] Zhang Y., Zhang S., Lin Y., Wu S., Li X., Yang C. (2025). Simultaneous Removal of Heavy Metals
and Antibiotics from Anaerobically
Digested Swine Wastewater via Functionalized Covalent Organic Frameworks. Environ. Res..

[44] Faiza, Hardacre B., Scott C., Winroth S., Ishida H. (2024). Synthesis of Bio-Based
and Intrinsically
Flame-Retardant Benzoxazine Containing Dynamic Ester Bond That Quantitatively
Satisfies All Twelve Principles of Green Chemistry. ACS Sustain Chem. Eng..

[45] Flood-Garibay J. A., Méndez-Rojas M. A. (2021). Synthesis
and Characterization of
Magnetic Wrinkled Mesoporous Silica Nanocomposites Containing Fe3O4
or CoFe2O4 Nanoparticles for Potential Biomedical Applications. Colloids Surf. A Physicochem Eng. Asp.

[46] Zych Ł., Osyczka A. M., Łacz A., Różycka A., Niemiec W., Rapacz-Kmita A., Dzierzkowska E., Stodolak-Zych E. (2021). How Surface Properties of Silica
Nanoparticles Influence
Structural, Microstructural and Biological Properties of Polymer Nanocomposites. Materials.

[47] Xie M., Xu L., Yang Z., Lin L., Wu K., Jiang Z. (2024). Nano-Enhanced
Asphalt Binders Incorporating Nanosilica-Grafted Carbon Nanofibers:
A Promising Route for Performance Improvement via Surface Modification. Constr Build Mater..

[48] Benyettou R., Amroune S., Slamani M., Saada K., Fouad H., Jawaid M., Sikdar S. (2023). Modelling
and Optimization of the
Absorption Rate of Date Palm Fiber Reinforced Composite Using Response
Surface Methodology. Alexandria Engineering
Journal.

[49] Zakir, M. ; Wolbring, G. ; Yanushkevich, S. A Causal Approach to Investigating Accessibility Experiences of Persons with Disabilities. In 2024 Sixth International Conference on Intelligent Computing in Data Sciences (ICDS); IEEE: 2024; pp 1–8.

[50] Li S., Sauber M. E., Etherington-Rivas M., Azimi G. (2024). Optimization of Supercritical
Fluid Extraction of Rare Earth Elements from Complex Ores Using a
Tributyl Phosphate-Nitric Acid Adduct. ACS Sustain
Chem. Eng..

[51] Abbas M., Trari M. (2024). Removal of Amoxicillin From Wastewater
Onto Activated Carbon: Optimization
of Analytical Parameters by Response Surface Methodology. Dose-Response.

[52] Sallam S., Alorabi A. Q., Almotairy A. R. Z., Ibarhiam S. F., Aljuhani E., Al-Qahtani S. D., El-Metwaly N. M. (2023). Superior and Effective Adsorption
of Amoxicillin by Using Novel Metal Organic Framework and Its Composite:
Thermodynamic, Kinetic, and Optimization by Box–Behnken Design. Appl. Organomet. Chem..

[53] Selim M. M., Tounsi A., Gomaa H., Hu N., Shenashen M. (2024). Addressing
Emerging Contaminants in Wastewater: Insights from Adsorption Isotherms
and Adsorbents: A Comprehensive Review. Alexandria
Engineering Journal.

[54] Beiranvand M., Farhadi S., Mohammadi-Gholami A. (2022). Adsorptive
Removal of Tetracycline
and Ciprofloxacin Drugs from Water by Using a Magnetic Rod-like Hydroxyapatite
and MIL-101­(Fe) Metal–Organic Framework Nanocomposite. RSC Adv..

[55] Jain G., Bhattacharyya P., Mandal M. K., Chaudhuri R. G., Chakrabarti S. (2024). PH-Dependent
Adsorption of the Sulfamethoxazole Antibiotic
on HKUST-1@CNS Nanocomposite Corroborating Efficiency, Mechanistic,
and Kinetic Studies. New J. Chem..

[56] Ragab A. H., Hussein H. S., Ahmed I. A., Abualnaja K. M., AlMasoud N. (2021). An Efficient Strategy for Enhancing
the Adsorption
of Antibiotics and Drugs from Aqueous Solutions Using an Effective
Limestone-Activated Carbon–Alginate Nanocomposite. Molecules.

[57] Mohammadi L., Rahdar A., Khaksefidi R., Ghamkhari A., Fytianos G., Kyzas G. Z. (2020). Polystyrene Magnetic
Nanocomposites
as Antibiotic Adsorbents. Polymers (Basel).

[58] Sadia M., Ahmad I., Aziz S., Khan R., Zahoor M., Ullah R., Ali E. A. (2024). Carbon-Supported
Nanocomposite Synthesis,
Characterization, and Application as an Efficient Adsorbent for Ciprofloxacin
and Amoxicillin. ACS Omega.

[59] Annu A., Mittal M., Tripathi S., Shin D. K. (2024). Biopolymeric Nanocomposites
for Wastewater Remediation: An Overview on Recent Progress and Challenges. Polymers (Basel).

[60] Elgarahy A. M., Elwakeel K. Z., Akhdhar A., Hamza M. F. (2021). Recent Advances
in Greenly Synthesized Nanoengineered Materials for Water/Wastewater
Remediation: An Overview. Nanotechnology for
Environmental Engineering.

[61] Zulfiqar N., Nadeem R., Musaimi O. A. (2024). Photocatalytic Degradation
of Antibiotics
via Exploitation of a Magnetic Nanocomposite: A Green Nanotechnology
Approach toward Drug-Contaminated Wastewater Reclamation. ACS Omega.

[62] Rawat S., Singh J. (2021). Green Synthesis of Iron Nanoparticles
Using Plumeria and Jatropha:
Characterization and Investigation of Their Adsorption. Regeneration and Catalytic Degradation Efficiencies. Bionanoscience.

[63] Rasoulzadeh H., Mohseni-Bandpei A., Hosseini M., Safari M. (2019). Mechanistic Investigation
of Ciprofloxacin Recovery by Magnetite–Imprinted Chitosan Nanocomposite:
Isotherm, Kinetic, Thermodynamic and Reusability Studies. Int. J. Biol. Macromol..

